# A multilayer perceptron neural network approach for optimizing solar irradiance forecasting in Central Africa with meteorological insights

**DOI:** 10.1038/s41598-024-54181-y

**Published:** 2024-02-12

**Authors:** Inoussah Moungnutou Mfetoum, Simon Koumi Ngoh, Reagan Jean Jacques Molu, Brice Félix Nde Kenfack, Raphaël Onguene, Serge Raoul Dzonde Naoussi, Jean Gaston Tamba, Mohit Bajaj, Milkias Berhanu

**Affiliations:** 1 Technologies and Applied Sciences Laboratory, University Institute of Technology of Douala, University of Douala, P.O. Box: 8689, Douala, Cameroon; 2 Department of Industrial Engineering and Maintenance, University Institute of Technology of Douala, University of Douala, P.O. Box: 8689, Douala, Cameroon; 3 Department of Thermal Engineering and Energy, University Institute of Technology of Douala, University of Douala, P.O. Box: 8689, Douala, Cameroon; 4 Transport and Applied Logistic Laboratory, University Institute of Technology of Douala, University of Douala, P.O. Box: 8689, Douala, Cameroon; 5 Department of Electrical Engineering, Graphic Era (Deemed to be University), Dehradun, 248002 India; 6 Hourani Center for Applied Scientific Research, Al-Ahliyya Amman University, Amman, Jordan; 7 Graphic Era Hill University, Dehradun, 248002 India; 8 Applied Science Research Center, Applied Science Private University, Amman, 11937 Jordan; 9 Department of Electrical and Computer Engineering, College of Engineering, Addis Ababa Science and Technology University, Addis Ababa, Ethiopia

**Keywords:** Solar radiation, Feed-forward network, Multilayer perceptron, Neural network, Energy science and technology, Engineering, Mathematics and computing

## Abstract

Promoting renewable energy sources, particularly in the solar industry, has the potential to address the energy shortfall in Central Africa. Nevertheless, a difficulty occurs due to the erratic characteristics of solar irradiance data, which is influenced by climatic fluctuations and challenging to regulate. The current investigation focuses on predicting solar irradiance on an inclined surface, taking into consideration the impact of climatic variables such as temperature, wind speed, humidity, and air pressure. The used methodology for this objective is Artificial Neural Network (ANN), and the inquiry is carried out in the metropolitan region of Douala. The data collection device used in this research is the meteorological station located at the IUT of Douala. This station was built as a component of the Douala sustainable city effort, in partnership with the CUD and the IRD. Data was collected at 30-min intervals for a duration of around 2 years, namely from January 17, 2019, to October 30, 2020. The aforementioned data has been saved in a database that underwent pre-processing in Excel and later employed MATLAB for the creation of the artificial neural network model. 80% of the available data was utilized for training the network, 15% was allotted for validation, and the remaining 5% was used for testing. Different combinations of input data were evaluated to ascertain their individual degrees of accuracy. The logistic Sigmoid function, with 50 hidden layer neurons, yielded a correlation coefficient of 98.883% between the observed and estimated sun irradiation. This function is suggested for evaluating the intensities of solar radiation at the place being researched and at other sites that have similar climatic conditions.

## Introduction

### Context

The development of human civilization is closely connected to the pursuit of harnessing energy, creating technical progress, achieving economic expansion, and promoting social well-being^1,2^. The transition from relying on human labor and biomass to the industrial revolution represented a significant change towards the use of non-renewable resources such as coal and steam power^3^. A significant portion of the global energy requirements in the present day is met by non-renewable fossil fuels, including coal, oil, and natural gas^4^. These fuels are used to power various sectors such as industry, transportation, and households. Nevertheless, this reliance encounters a significant obstacle—the limited availability of fossil fuel resources^5,6^. The limited stock of fossil fuels is a significant obstacle, considering the increasing world population and industrialization^7^. The increasing demand of energy brings to legitimate worries regarding the sustainability of current energy infrastructures, due to the undeniable fact that fossil fuel sources are finite^8^. Due to the diminishing stocks of fossil fuels and growing environmental concerns, there is a significant movement towards alternate and renewable energy sources. In addition to technological factors, this transition is a worldwide necessity motivated by the pressing requirement for sustainability and the preservation of the environment^9,10^. The increasing worldwide energy demand, along with the recognition of the depletion of conventional energy sources, necessitates a crucial reassessment of our energy situation^11^. The need to reduce environmental deterioration is in line with the objectives of ecological stewardship, representing a crucial point where energy transitions connect with wider ecological resilience^12^. Our research adds to the discussion on sustainable energy practices, offering valuable insights for managing the challenges of shifting towards renewable and ecologically friendly energy sources. Given the historical development of energy sources, the present difficulties, and the urgent need for sustainable options, our study becomes important and pertinent in tackling modern energy challenges^13,14^.

Central Africa's ongoing energy shortfall has been a major roadblock to long-term economic growth and sustainable development. Urgent and creative solutions are needed to address the region's significant dependence on old fossil fuel-based energy sources and the rising demand for power. Despite these obstacles, accurate predictions of solar irradiance are becoming more important for a sustainable energy plan^15,16^. The region's energy infrastructure may be made more stable and resilient with the help of accurate projections of solar irradiance, which can improve the efficiency and dependability of solar energy production^17^.

The critical need to tackle the energy shortage in Central Africa has sparked a growing emphasis on the promotion of renewable energy sources as a possible solution. Extensive academic study highlights the significant solar energy potential in Cameroon, emphasizing its crucial role in the sustainable energy development of the area. The detailed understanding of changes in solar output becomes particularly important in the careful design of renewable energy conversion systems. This comprehensive understanding plays a crucial role in directing decisions in several aspects of system engineering, including intricate design, optimal size, rigorous performance assessments, and judicious energy management techniques^18,19^.

The necessity of solar photovoltaic power in defining the future of sustainable energy is undeniable^20^. The Earth's most plentiful energy source, solar radiation, has the ability to bring about significant transformation. Reliable sources, such as^21^, emphasize the remarkable fact that the amount of solar energy that the Earth absorbs from the sun in a single hour is sufficient to fulfill the world's energy requirements for an entire year. To fully exploit this potential, it is crucial to have a detailed comprehension of how solar radiation is distributed at specific installation sites. This requires careful analysis of various orientations and inclinations in order to maximize the efficiency of photovoltaic collectors^22,23^. Estimating solar irradiance is a challenging task influenced by several geographical and astronomical factors. Additionally, the constant interaction of meteorological and atmospheric conditions further complicates this job. In spite of these difficulties, several estimating methods have arisen, utilizing meteorological data at different time intervals—hourly, daily, and monthly—to improve accuracy in forecasting solar irradiation^24,25^. This comprehensive strategy emphasizes the collaborative endeavors to fully use the potential of solar energy and enhance the efficiency of photovoltaic systems in order to fulfill the growing global energy needs^26^.

### Review of the literature

**Figure 1:**
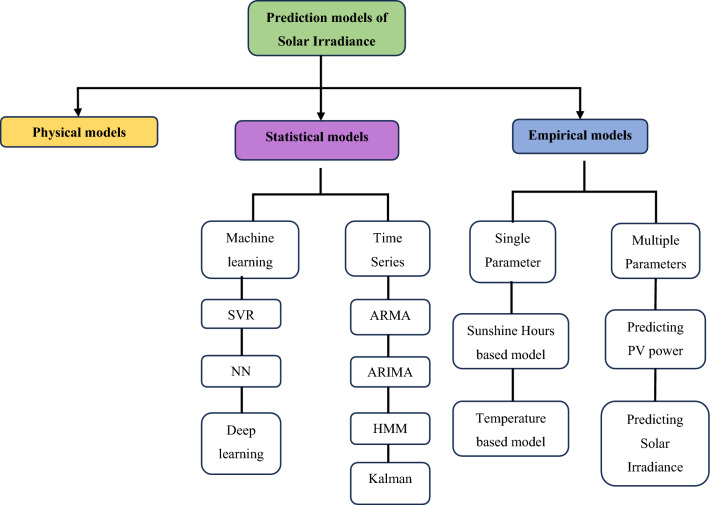
Solar irradiance prediction methods^18^.

The prediction of solar irradiance is of utmost importance in the field of renewable energy generation, as explained by authoritative sources^27^. Forecasting has the capacity to greatly improve the effectiveness of planning and operating photovoltaic systems, leading to numerous economic benefits for electric utilities^28,29^. The increasing use of renewable energy, motivated by the unpredictable nature of fossil fuel costs, worries about public well-being, and a heightened global understanding of climate change, emphasizes the need for improvements in the current power grid^30,31^. The utilization of renewable energy sources offers both economic benefits and social advantages. Nevertheless, the incorporation of these sources into the power grid is frequently hindered by obstacles such as irregularity^32^. Forecasting techniques are valuable tools that can address this issue by providing insights into upcoming patterns and enabling users to make well-informed decisions ahead of time. Figure 1, as described in the literature^27^, illustrates the common methods used for predicting solar irradiance. It visually presents the various approaches used to address the challenges of integrating renewable energy.

Quantitative forecasting methods offer a viable means to depict the time series of solar irradiance, utilizing historical data to predict forthcoming samples, as delineated in the literature^33,34^. The replicability of mean solar irradiance can be posited, with a consistent trend of monthly mean solar irradiance exceeding during the dry season compared to the rainy season^35^. The temporal evolution of solar irradiance, per existing literature, manifests stochastic behavior intricately tied to cloud movement dynamics^27,36^. This nuanced interplay underscores the complexity inherent in modeling solar irradiance, a task broadly categorized into three distinct groups: physical, statistical, and empirical models^37^. These modeling approaches collectively contribute to a comprehensive understanding of solar irradiance patterns, encompassing both deterministic and probabilistic factors, and are essential for informed decision-making in the realm of solar energy applications^38,39^.

#### Models Physics

In the literature, a number of physical models for estimating solar irradiation may be discovered. The irradiance model developed by^40–42^ is one of the most used physical models. The following Eq. (1) illustrates how the total irradiance is the sum of the direct, diffuse, and ground-reflected irradiances:

1 $${{\text{G}}}_{{\text{TOT}}}={{\text{G}}}_{{\text{DNI}}}.{\text{cos}}\left(\uptheta \right)+{{\text{G}}}_{{\text{DIFF}}}\left(\frac{1+{\text{cos}}\left(\upbeta \right)}{2}\right)+{{\text{G}}}_{{\text{REFL}}}\left(\frac{1-{\text{cos}}\left(\upbeta \right)}{2}\right)$$

The PV panel's tilt angle is represented by *β*, while the solar angle is denoted by *θ*. The calculation of the solar angle can be performed using Eq. (2).

2 $${\text{cos}}\left(\uptheta \right)={\text{cos}}\left({\text{z}}\right){\text{cos}}\left(\upbeta \right)+{\text{sin}}\left({\text{z}}\right){\text{sin}}\left(\upbeta \right){\text{sin}}\left({{\varnothing }}_{{\text{s}}}-\upgamma \right)$$

**Figure 2:**
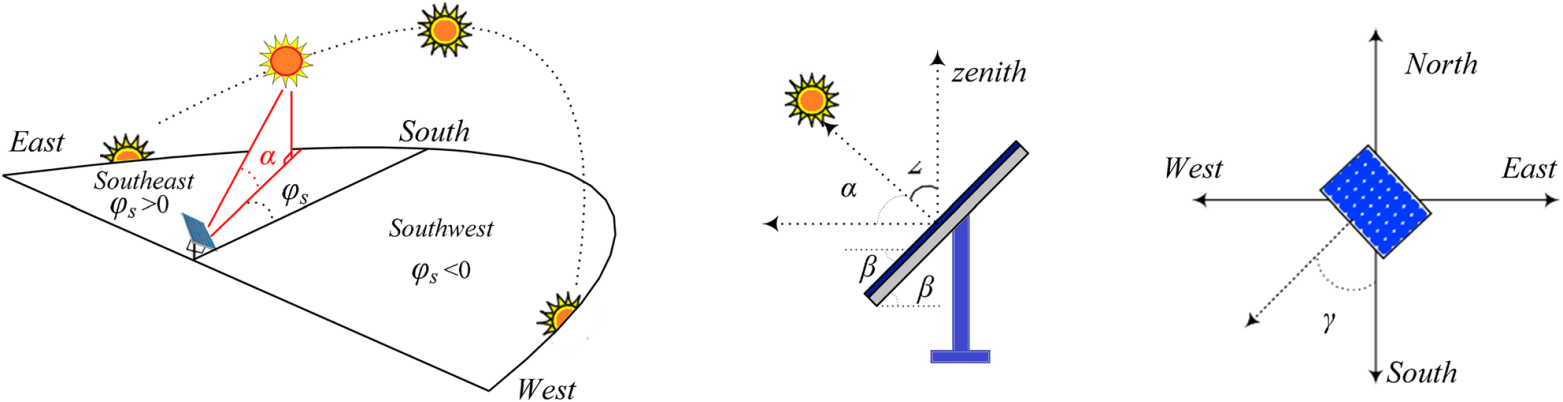
Characteristic of the solar angle^21^.

The angle at zenith, denoted by z, is defined as the angle between the vertical line and the beam radiation. The solar azimuth angle, denoted by ${{\varnothing }}_{{\text{s}}}$, is defined as the angle between the south of the beam projection and the PV surface. The solar angles' characteristics are depicted in Fig. 2**.**

#### The statistical models

Numerous statistical models have been documented in the literature for the purpose of predicting solar irradiance^43,44^. Statistical models can be classified into two categories: time series methods, which include ARMA, ARIMA, and HMM, and machine learning algorithms, such as Neural Networks, Deep learning, and RVS^45^. Solar irradiance is a time series that comprises three components in time series methods. These components include the long-term trend, the periodic components, and the mean. The ARMA (p, q) autoregressive moving average^46–48^ is the predominant technique for time series prediction. It can be represented by Eq. (3):

3 $${{\text{y}}}_{{\text{t}}}={\text{c}}+{{\varnothing }}_{1}{{\text{y}}}_{{\text{t}}-1}+\dots +{{\varnothing }}_{{\text{p}}}{{\text{y}}}_{{\text{t}}-{\text{b}}}+{\upvarepsilon }_{{\text{t}}}+{{\varnothing }}_{2}{\upvarepsilon }_{{\text{t}}-2}+\dots {\uptheta }_{2}{\upvarepsilon }_{{\text{t}}-2}+\dots +{{\varnothing }}_{{\text{q}}}{{\text{y}}}_{{\text{t}}-{\text{q}}}+{\upvarepsilon }_{{\text{t}}}$$

Equation (3) comprises of two components: an autoregressive (AR) component in the first part and a moving average (MA) component in the second part. The Yule-Walker method can be utilized to identify the variables. Prior to the application of this approach, it is necessary to conduct a stationarity test on the time series. This requirement may be considered a disadvantage of time series prediction techniques. Machine learning techniques have gained widespread adoption. The prevalent approach for machine learning is the Support Vector Machine (SVM), which is a supervised learning algorithm. SVM is a viable computational tool for the purpose of prediction and classification. SVM utilizes the concept of decision planes to establish decision boundaries^46^. SVM has been utilized in the field of forecasting. However, it may not possess the capability to extract long-term correlations from time series or very short-term components.

#### Empirical models

Various models are present in the academic literature. The predominant approach entails employing a sunshine-based model, as expressed in Eq. (4), wherein a and b represent empirical coefficients, and GHI denotes the monthly average of global horizontal irradiance. The variable S represents the mean monthly duration of sunlight. The duration of daylight is indicated by references So^12,47,48^:

4 $$\frac{{{\text{GHI}}}_{{\text{avg}}}}{{{\text{H}}}_{0}}={\text{a}}+{\text{b}}\frac{{\text{S}}}{{{\text{S}}}_{0}}$$

### Neural network

#### Feedforward neural network (FFNN)

**Figure 3:**
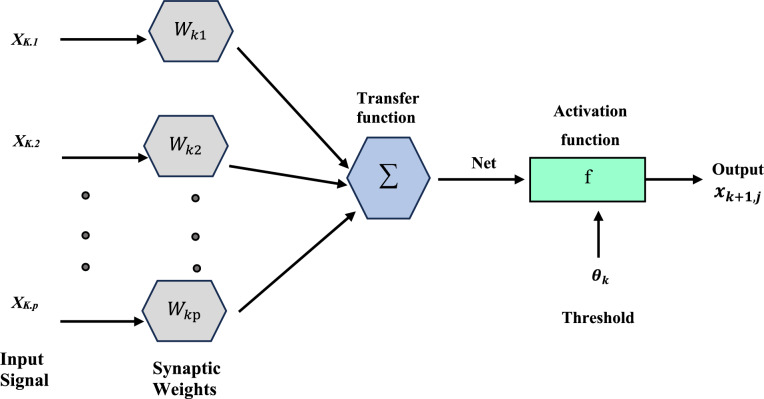
Simple feedforward neural network.

The first form of the artificial neural network created was the feedforward neural network (FFNN). Neural networks are a kind of computer technique that use a large number of simulated neurons^49,50^. These nerve cells are roughly analogous to an axon in a real brain. Machine learning, image processing, signal processing, computer science, controlling power electronics converters that interact with PV systems, and modeling energy sources are just some of the many applications for neural networks^51–53^. A neuron, an activation function, and a bias make up these components. A neuron's function may be either as an input, an output, or a hidden neuron. A basic hidden-layers forward-propagation neural network is shown in Fig. 3^53,54^.

#### Recurrent neural network (RNN)

**Figure 4:**
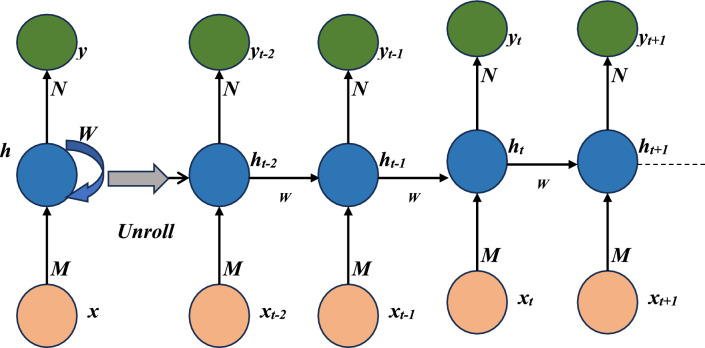
RNN unfolded (left), and RNN folded (right).

The output of a recurrent neural network is dependent on the input, making it useful for modeling and predicting sequential data^55^. Image analysis, emotion detection, language translation, and voice recognition are just a few of the uses that have made use of this technology^56^. The RNN can use its own memory to make predictions about future inputs, even if those inputs are completely random. The results of a prior computation may be stored in the device's internal memory^57^. The fundamental RNN is seen in Fig. 4; in this architecture, the hidden neuron *h* gets weighted feedback from other neurons in the preceding time step. When the basic RNN is expanded into a full network, it becomes clear that each neuron's input is fed by the outputs of neurons in the preceding time step^54^.

The input of the first hidden neuron is calculated by multiplying the input* x*_*t*_ at time *t* by the input weight vector. The output of *x*_*t*+1_ is multiplied by the weight W of the previous hidden neuron *h*_*t*_ to form the input of the following hidden neuron *h*_*t*+1_. Only the hidden neurons, multiplied by the output weight *N*, are used to feed the output neurons. The system's dynamics are described by the following Eqs. (5) and (6):

5 $${{\text{h}}}_{{\text{t}}}={{\text{f}}}_{{\text{h}}}\left({\text{M}}*{{\text{x}}}_{{\text{t}}}+{\text{W}}+{{\text{h}}}_{{\text{i}}-1}\right)$$

6 $${{\text{y}}}_{{\text{t}}}={{\text{f}}}_{{\text{y}}}\left({\text{N}}*{{\text{h}}}_{{\text{t}}}\right)$$

where *f* represents a specific activation function like sigmoid, tanh, or ReLU. Backpropagation over time (BPT) is a technique similar to backpropagation (BP) that is used to train artificial neural networks. The difference between BP and BPT is that BPT considers both the present and historical states.

#### Artificial neural networks

**Figure 5:**
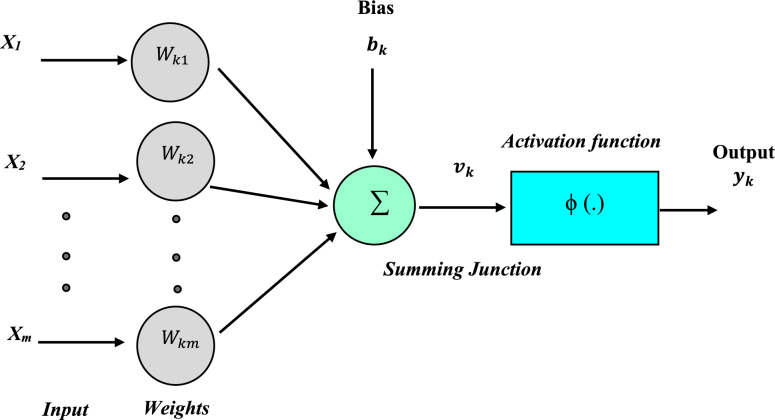
Simple structure of ANN.

Among the many applications for artificial neural networks (ANNs) include prediction, curve-fitting pattern recognition, simulation, regression, optimization, modeling, clustering, simulation, and more^58^. Models for predicting solar radiation are formulated with the help of artificial neural networks in this study^59^. A neuron, the basic building block of artificial neural networks, processes input data using a transfer function to generate an output. Weights multiply each input, representing the connection between the input and the neuron and also between the several layers of neurons. At last, the neuron employs a transfer function to get the answer. The ANN's overarching structure is seen in Fig. 5. In comparison to other methods, ANN procedures use less processing work and provide a more condensed answer to problems with several variables, all while eliminating the requirement for experts to know how to do mathematical computations between the parameters^60^.

Researchers in the area of renewable energy have taken notice of artificial neural networks, in particular for the prediction of meteorological data like solar irradiance.

Koumi et al.^61^ used a feed-forward backpropagation artificial neural network to estimate the solar radiation in the city of Garoua (9.3 N, 13.4 E, altitude: 242 m). From 1995 through 2003, he collected this information from a NASA geo-satellite. It used as input the amount of time the sun was out, the relative humidity, the temperature, and the barometric pressure. The greatest results he got were an MBE and RMSE that were close to zero and a deterministic coefficient of about 98%. A comprehensive set of meteorological data including average sunlight duration, average temperature, pressure, and relative humidity is required for accurate solar radiation predictions using ANN, as shown by the results of the performed comparison research.

The authors Voyant et al.^62^ have conducted research on utilizing artificial neural networks (ANNs) to predict time series data for solar radiation and photovoltaic energy production. The purpose of this research is to accurately measure the available energy and facilitate efficient management of the transition between intermittent and conventional energy sources. In this study, various prediction techniques were evaluated on four horizons that are typically relevant to network managers. These horizons include *d* + 1, *h* + 24, *h* + 1, and *m* + 5. Upon completion of the aforementioned manipulations, it has been determined that the hierarchy of the various predictors varies depending on the considered horizon. Utilizing a neural network methodology for the horizon *j* + 1 can be a compelling option. It is important to ensure that the time series is stationary and that exogenous variables are incorporated.

In the field of predicting sunshine data using artificial intelligence methods, specifically ANN, Kalogirou^63^, Kalogirou et al.^64^, and Benghanem^65^ have provided a comprehensive overview of the current state of the art. The following methods were reviewed: ANN, Fuzzy Logic, Genetic Algorithms, Expert Systems, and Hybrid Methods. The articles presented here primarily serve as reference materials within the domain of Sunshine forecasting. In their study, Hontoria et al.^66^ presented a Multilayer Perceptron Artificial Neural Network (MLP-ANN) as a means of producing synthetic solar irradiation series. The Multilayer Perceptron (MLP) was compared to two other conventional methods, and it demonstrated superior performance compared to the other two methods. Hontoria et al.^67^ utilized a recurrent artificial neural network (ANN) to accurately model the hourly solar irradiance across Spain. Gazala et al.^68^ utilized a back-propagation artificial neural network to analyze solar irradiance data in Athens spanning from 1997 to 1999. The data included both horizontal and tilted measurements. The study was conducted during both the winter and summer seasons. The proposed neural network architecture referred to as ANN comprises 6 hidden neurons for summer and 10 hidden neurons for winter. The ANN model is designed to predict the tilted solar irradiance at time t based on the horizontal irradiations at time *t* and (*t*−1), as well as the tilted solar irradiance at the time (*t* + 1). Mellit et al.^69^ employed ANN to forecast 24-h solar irradiance based on the daily average solar irradiance and air temperature. The data were collected through measurements taken in Trieste, Italy over a span of 14 months. Several configurations of multilayer perceptron were evaluated, and the optimal configuration consists of three input neurons, two hidden layers with 11 and 17 neurons, respectively, and 24 output neurons. The aforementioned method can be conveniently modified to forecast solar irradiance for the next 24 h. This can be achieved by incorporating various inputs such as cloud cover, pressure, wind speed, sunshine duration, and geographical coordinates.

A comparative study of various artificial intelligence algorithms was conducted by Premalatha Neelamegam et al.^70^. The objective of this study was to develop an ANN model for the purpose of predicting the monthly average solar radiation in India. The study involved the training and testing of two distinct artificial neural network models, each utilizing four backpropagation algorithms, namely gradient descent (GD), Levenberg–Marquardt (LM), scaled conjugate gradient (SCG), and resilient backpropagation (RP) algorithm. Over a span of 10 years, meteorological data was gathered from five stations across India's geography. This data was utilized to train and test the network.

ANN was utilized by Zahraa E. Mohamed^71^, to forecast solar irradiance in various cities located in Egypt. This study employed ANN-based models to assess and forecast solar irradiance for three cities located in Egypt. Based on the statistical indicators, it has been determined that the second algorithm outperforms the other artificial neural network models when tested with the data. Furthermore, it can be observed that R2 values exceed 99% in every instance, and the corresponding RMSE values are minimal. The results demonstrate that the Bp algorithm, when combined with momentum and a specific learning rate, outperforms the baseline Bp algorithm. Additionally, the second algorithm exhibits the highest level of performance among all cities. The findings indicate that the developed ANN model may serve as a superior substitute for conventional estimation models while maintaining an acceptable level of accuracy. Benatiallah et al.^72^ utilized a combination of two techniques, namely artificial neural network and fuzzy logic, to estimate solar irradiance. This hybrid approach is referred to as "neuro-fuzzy" in their research. The author demonstrated the reliability of this approach in situations where the available input data is insufficient.

The objective of this study was to develop an artificial neural network (ANN) model for the prediction of monthly average global solar radiation values in India. The model was trained using meteorological data collected over a period of 24 months from Douala, Cameroon. The proposed methodology has an advantage in that it allows for implicit utilization of problem-associated information, without requiring prior knowledge of the correlation between solar irradiance and the different variables.

The objective of this research is to create a neural model capable of forecasting the daily monthly average solar irradiance received on a slope in Douala. The anticipated data has the potential to aid in the sizing of a photovoltaic system. The study will employ the neural approach Artificial Neural Network methodology to examine the impact of the aforementioned parameters and evaluate the irradiance's sensitivity to each variable. The variables will undergo modeling and simulation processes utilizing the toolboxes available in MATLAB software.

The prediction of solar irradiance is a crucial subject in the field of renewable energy generation. The utilization of prediction techniques enhances the efficiency of planning and operation of photovoltaic systems, thereby providing significant economic advantages to electric utilities. One potential solution to address the energy deficit in Central Africa is the promotion of renewable energy sources.

### Research gaps and study contributions

The following points illustrate the originality and depth of this study's contributions to the field of knowledge.

- The study employs an Artificial Neural Network (ANN) as the technique for forecasting solar irradiation on a tilted surface. Although artificial neural networks (ANN) are often used, this is the first instance of its use in predicting solar radiation while considering the influence of climatic conditions. This showcases the innovative use of machine learning techniques to tackle the problem of forecasting solar irradiation in the region.
- The research incorporates weather factors that impact solar radiation. The circumstances include temperature, wind velocity, relative humidity, and atmospheric pressure. The research incorporates several parameters into the prediction model to handle the complexity of solar irradiance data and give a more comprehensive and accurate forecasting technique.
- This research focuses on the metropolitan area of Douala, Cameroon. A more precise prediction model may be constructed by narrowing the emphasis to a smaller geographical region. Having access to data and insights relevant to Douala is valuable for designing renewable energy projects in Central Africa, since the local climate patterns and solar irradiance characteristics may vary across different locations.
- Data was collected longitudinally from January 17, 2019, to October 30, 2020, with measurements of solar irradiance conducted at 30-min intervals. The extended duration for data collection enhances the accuracy of the prediction model by enabling the inclusion of seasonal variations and long-term patterns.
- The research used a systematic validation and testing approach by training the neural network using 80% of the data, confirming it with 15%, and testing it with the remaining 5%. By adhering to this rigorous approach, we ensure precise assessment of performance and mitigate the risk of model overfitting.
- The optimal neural network architecture for computing solar radiation intensities was determined to be a logistic Sigmoid function with 50 hidden layer neurons, according to the findings of the researchers. This finding offers novel insights into the most effective arrangement of neural networks for tasks related to forecasting solar radiation under comparable environmental conditions.
- The model exhibits a strong correlation coefficient of 98.883% between the observed and anticipated solar irradiance. Significant levels of correlation, such as the one shown, provide strong evidence of the effectiveness of the neural approach and are advantageous for strategizing and implementing solar energy programs.

## Methodology

**Figure 6:**
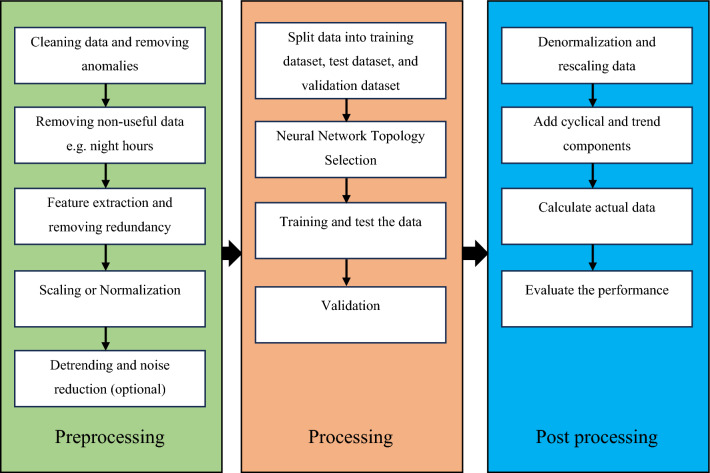
An approach to time series prediction.

Figure 6 shows the multiple processes involved in the prediction process.

Douala, Cameroon's solar potential is studied in detail by following these procedures.

### Collecting data

The metrological station at the IUT in Douala serves as the experimental prototype for our research. Douala is a seaside city that sits on the Wouri stream, 13 m above sea level, between 4°3′53.77 N of the equator and 9°41′15.41 E of the Greenwich meridian.

**Figure 7:**
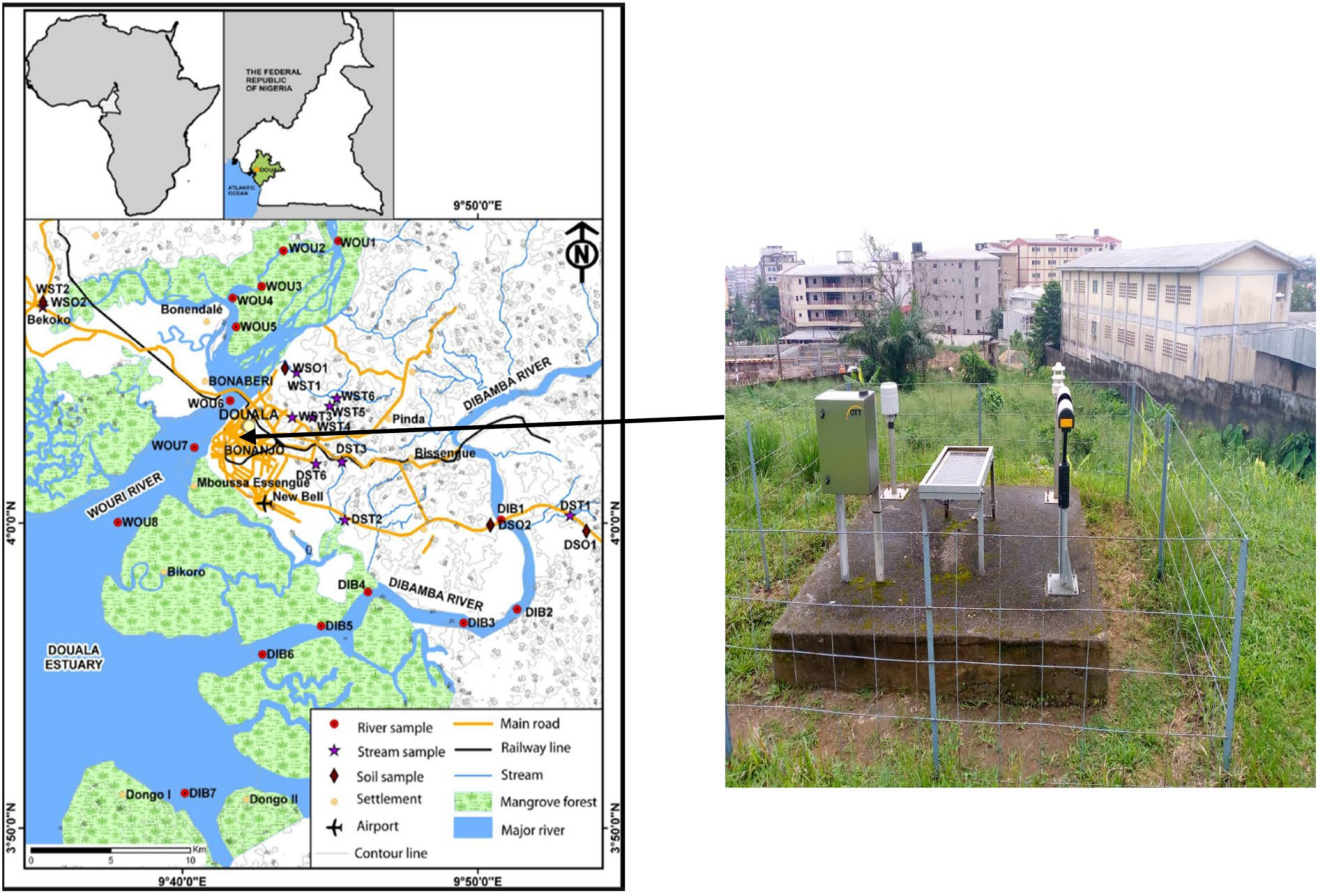
Meteorological station IUT Douala.

This meteorological station, seen in Fig. 7, is the source of the data. To better understand rainfall data and the hydrological regime of the drains in the Tongo Bassa catchment area, this station was set up as part of a collaborative effort between the Douala Institute of Technology (IUT) and the Institute of Research and Development (IRD). This effort is articulated through the Douala Sustainable City Project, which is funded by the CUD, the French Fund for the World Environment, and the French Development Agency. Solar irradiance (W/m2), temperature (°C), wind speed (m/s), relative humidity (%), and barometric pressure (Pa) are only a few examples of such data. For now, we have data from January 17, 2019, through October 30, 2020, a span of over two years gathered at 30-min intervals.

The different components of this station are:

- OTT Parsivel for precipitation measurement
- The OTT TRH weather sensor is compact and durable. Its WS range consists of:
  - An ultrasonic anemometer with electronic compass
  - A temperature sensor ;
  - A capacitive relative humidity sensor;
  - A barometric pressure sensor ;
  - A global radiation sensor (CMP3) ;
  - A liquid precipitation sensor by means of a tilting trough system;
  - A precipitation sensor LAMBRECH.

### Data analysis

**Figure 8:**
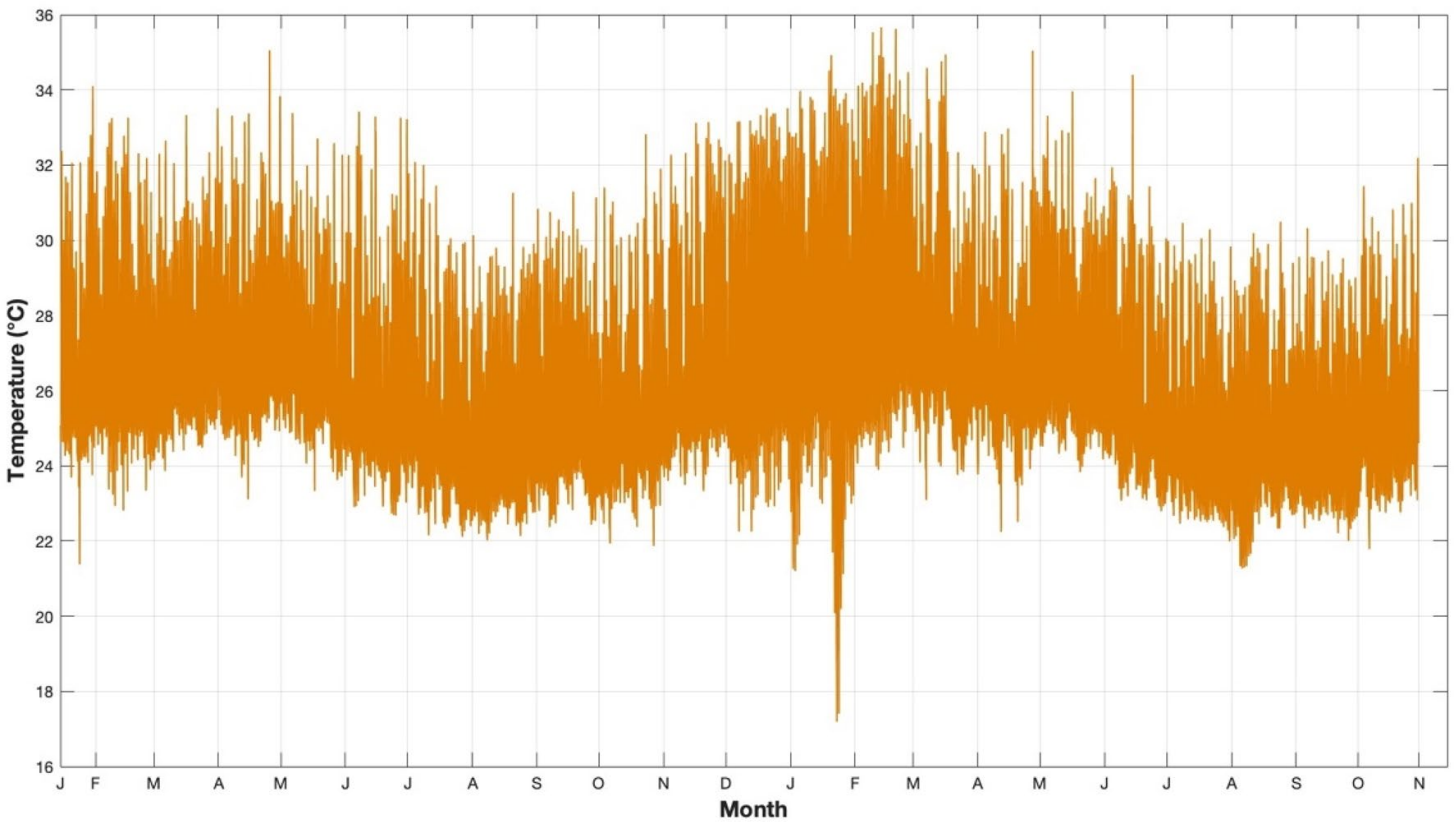
Temperature measurements in 30-min steps from January 17, 2019 to October 30, 2020.

**Figure 9:**
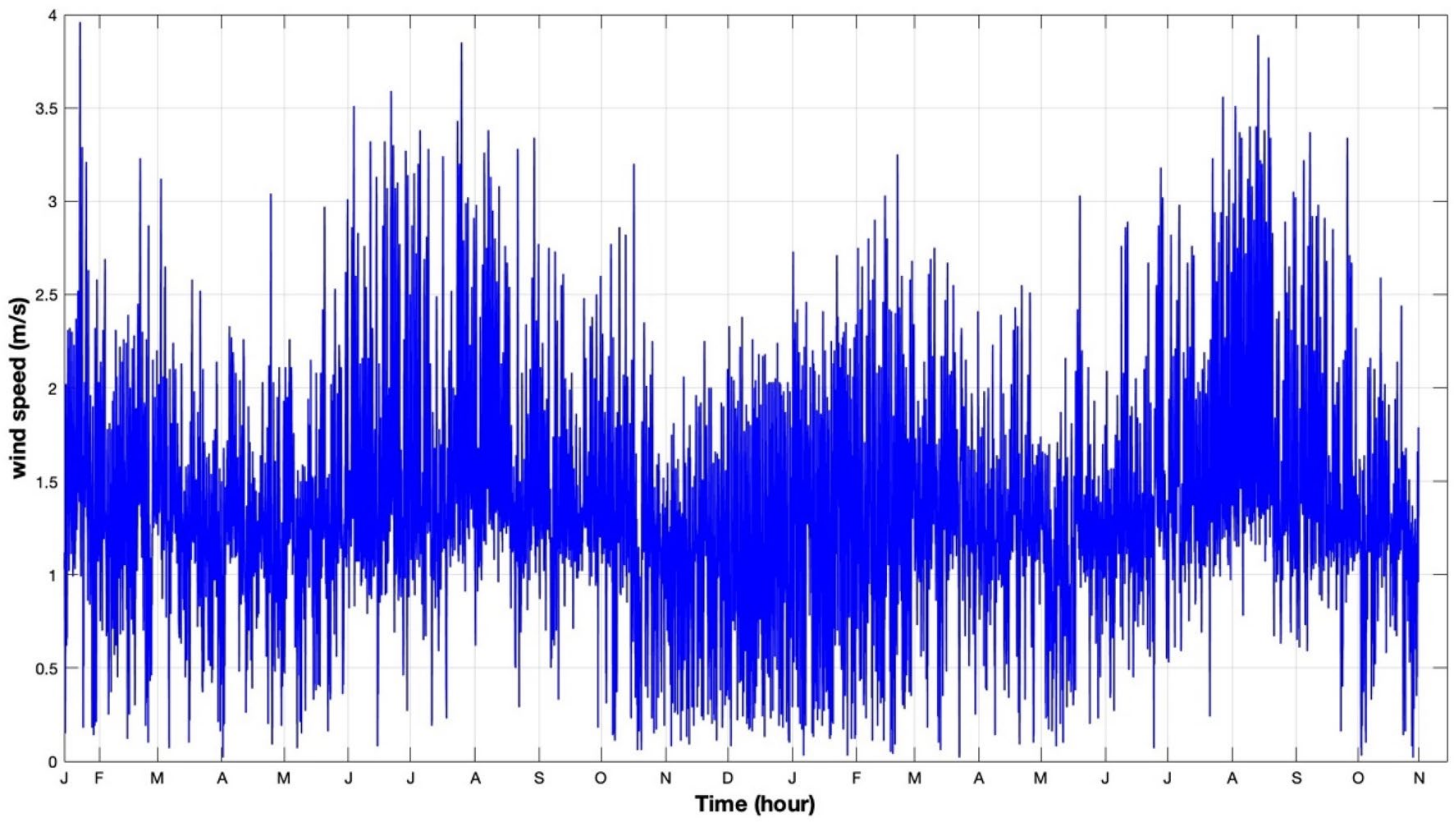
Wind speed measurements at 30-min intervals from January 17, 2019 to October 30, 2020.

**Figure 10:**
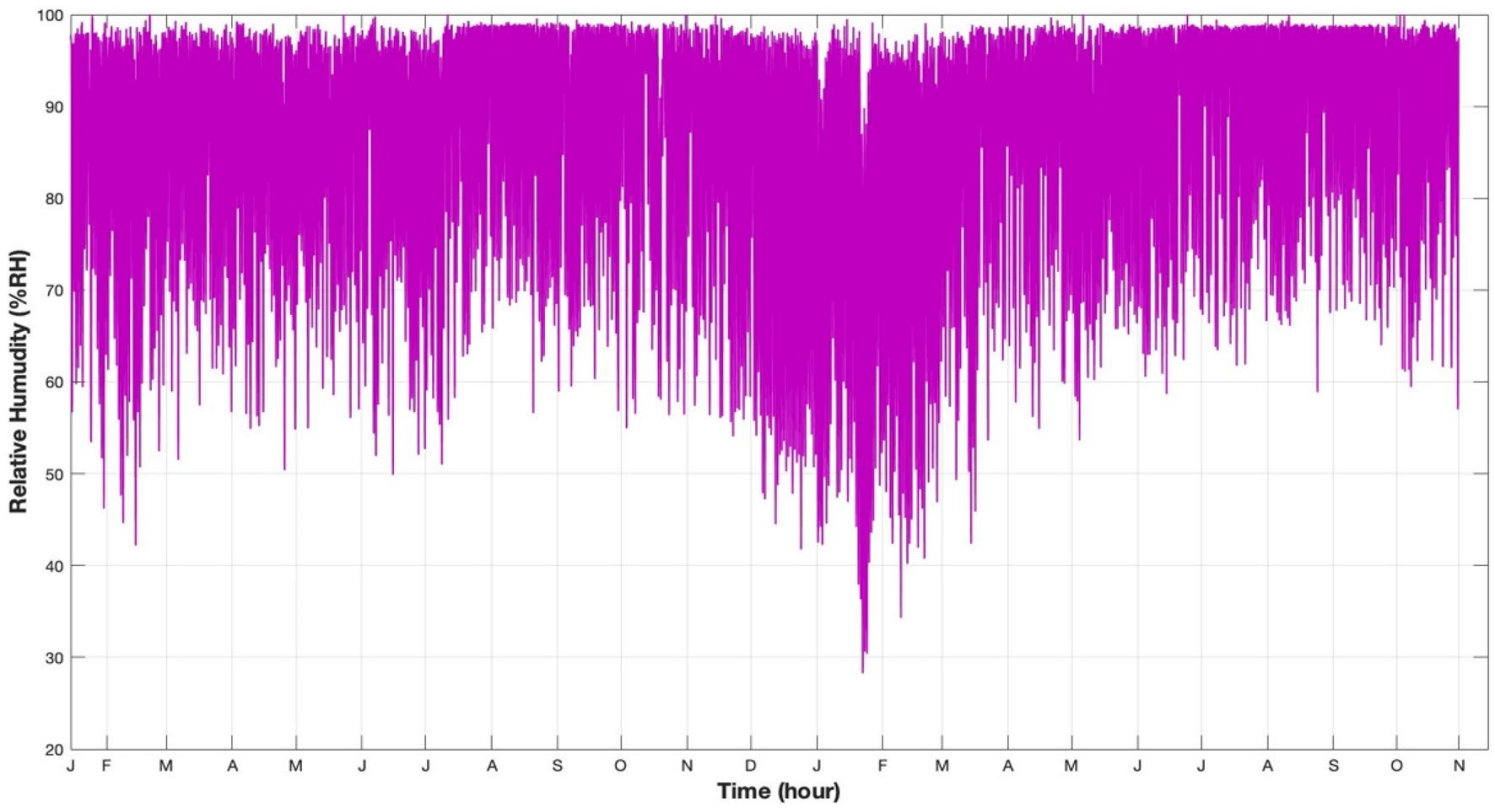
Relative humidity measurement in 30-min steps from January 17, 2019 to October 30, 2020.

**Figure 11:**
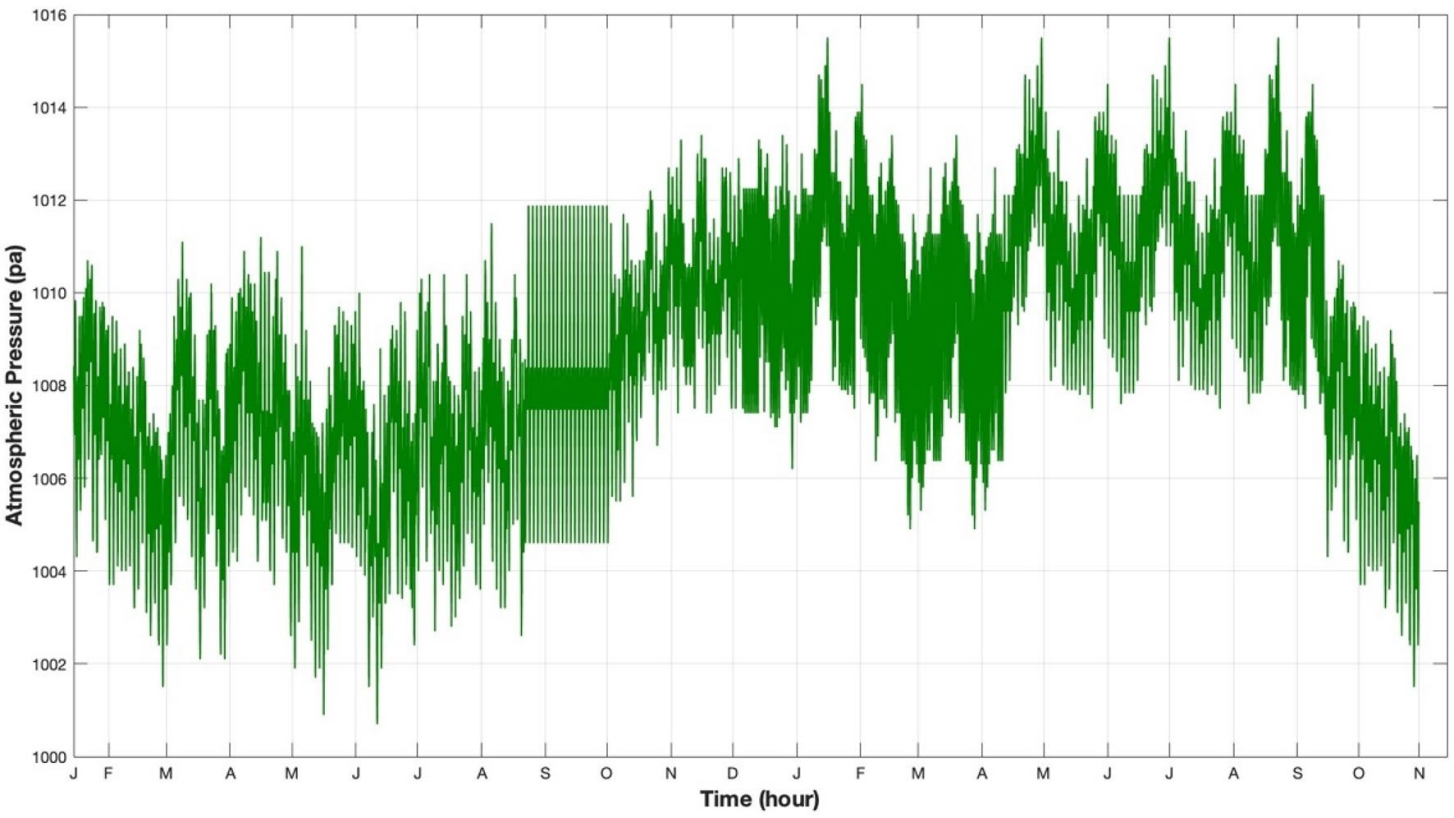
Atmospheric pressure measurements at 30-min intervals from January 17, 2019 to October 30, 2020.

Initially, this information is recorded in a database. Subsequently, the data is extracted, filtered, and analyzed using Excel. Excel is very beneficial in this specific situation due to its capacity to effectively generate, arrange, and sort data utilizing Dynamic Cross Tabulation (DST) techniques. Mean temperature, wind speed, relative humidity, and atmospheric pressure are shown in a time series plot in Figs. 8, 9, 10 and 11, respectively. There is a discernible seasonal component across all yearly plots. The four weather predictors may be compared to the output variable with the assistance of these charts.

## ANN-based methodology for Solar radiation prediction

The information processing in biological neural networks served as inspiration for a class of separate mathematical models known as artificial neural networks (ANNs), many of which are useful for predicting tasks and modeling nonlinear functions f. One of the features of ANNs is their ability to pick up information from their environment and improve their performance via a variety of learning mechanisms. Learning may be seen as a shift in the synaptic weights connecting neurons across neural layers. The goal is to make measured and predicted production numbers consistent with one another. Therefore, it is essential to choose the learning approach and to define the percentage of the whole data set that will be utilized for training. Selecting consists of the several processes required to create an optimal ANN^73^: input data; ANN architecture; transfer function; ANN size (number of layers; the number of neurons per layer); ANN learning technique; the ratio of training to testing data; ANN size.

### ANN structure

**Figure 12:**
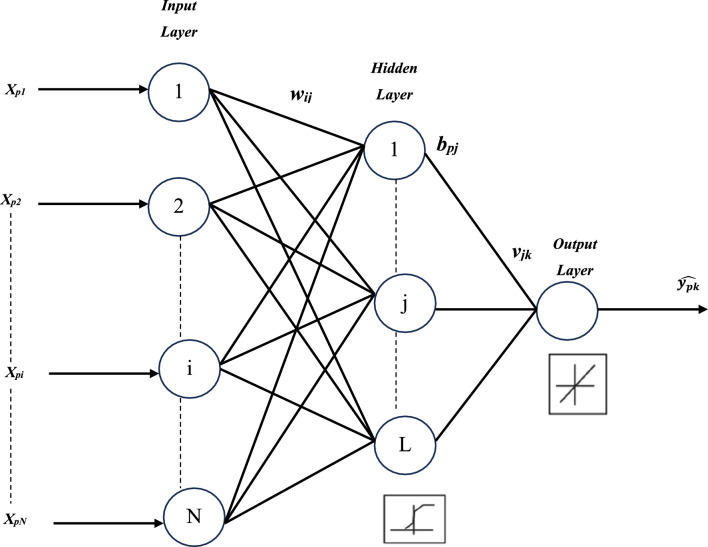
Multilayer perceptron.

A Multilayer Perceptron (MLP) was employed utilizing Feedforward back-propagation. The aforementioned structure is widely utilized in literature to estimate solar radiation and has demonstrated superior performance. According to Berke Akkaya^74^, the Multi-layer Perceptron exhibits several benefits in making complex predictions using non-linear data in the field of artificial intelligence. The system is comprised of three distinct layers, as outlined in reference^75^: the input layer, the output layer, and the hidden layer (refer to Fig. 12). The Multilayer Perceptron (MLP) is a non-recurrent Artificial Neural Network (ANN) paradigm that has been extensively researched. It provides a high degree of flexibility in forecasting due to its ability to accommodate varying numbers of input and output variables^76–79^. Multi-layer perceptrons (MLPs) provide significant flexibility in the design of forecasting models. Equation (7) defines the mathematical representation of the function that is applied by the hidden neurons to obtain an output value ${{\text{b}}}_{{\text{pj}}}$, when presented with an input vector or pattern ${{\text{X}}}_{{\text{P}}}$, which consists of ${{\text{X}}}_{{\text{P}}}$: ${{\text{x}}}_{{\text{p}}1},\dots , {{\text{x}}}_{{\text{pi}}}, \dots , {{\text{x}}}_{{\text{pN}}}$,.

7 $${{\text{b}}}_{{\text{pj}}}={{\text{f}}}_{{\text{L}}}\left({\uptheta }_{{\text{j}}}+\sum_{{\text{i}}=1}^{{\text{N}}}{{\text{w}}}_{{\text{ij}}}.{{\text{x}}}_{{\text{pj}}}\right)$$

The activation function of hidden neurons *L* is denoted as ${f}_{L}$. The threshold of hidden neuron j is represented by ${\uptheta }_{{\text{j}}}$, while w_ij_ denotes the weight of the connection between input neuron *i* and hidden neuron *j*. Lastly, *x*_*pi*_ refers to the input signal received by input neuron* i* for pattern* p*.

The output neurons' output is computed using Eq. (8), which is the same method used for calculating the hidden layer neurons' output.

8 $$\widehat{{{\text{y}}}_{{\text{pk}}}}={{\text{f}}}_{{\text{M}}}\left({\uptheta }_{{\text{k}}}+\sum_{{\text{j}}=1}^{{\text{L}}}{{\text{v}}}_{{\text{jk}}}.{{\text{b}}}_{{\text{pj}}}\right)$$

where $\widehat{{{\text{y}}}_{{\text{pk}}}}$ is the output signal given by output neuron* k* for pattern *p*, ${{\text{f}}}_{{\text{M}}}$ is the activation function of output neurons *M*, *k* is the threshold of output neuron *k*, and ${{\text{v}}}_{{\text{jk}}}$ is the weight of the link between hidden neuron *j* and output neuron *k*.

### ANN transfer function

The input layer neurons in this study do not employ transfer functions. However, the hidden layers utilize neurons with sigmoidal tangent (tansig) transfer functions, while the output layer employs neurons with linear (purlin) transfer functions. The neural network employs a sigmoid function, as defined in Eq. (9), in the hidden layer neurons to enable the learning of nonlinear functions. On the other hand, the output neuron uses a linear function, as defined in Eq. (10), to estimate continuous variables.

9 $${\text{f}}\left({\text{x}}\right)=\frac{1}{1+{{\text{e}}}^{-{\text{x}}}}$$

10 $${\text{f}}\left({\text{x}}\right)={\text{x}}$$

where x is aa variable.

### Learning algorithm

The Artificial Neural Network (ANN) model was trained using the Lavenberg-Marquardt backpropagation algorithm (LM algorithm). The Levenberg–Marquardt (LM) algorithm is a modified version of Newton's method that exhibits superior performance when applied to time series and transient series. The algorithm in question offers a balanced solution between the rapidity of Newton's method and the assured convergence of the steepest descent algorithm, as stated in references^68,78^.

### Training, validating, and test set

**Figure 13:**
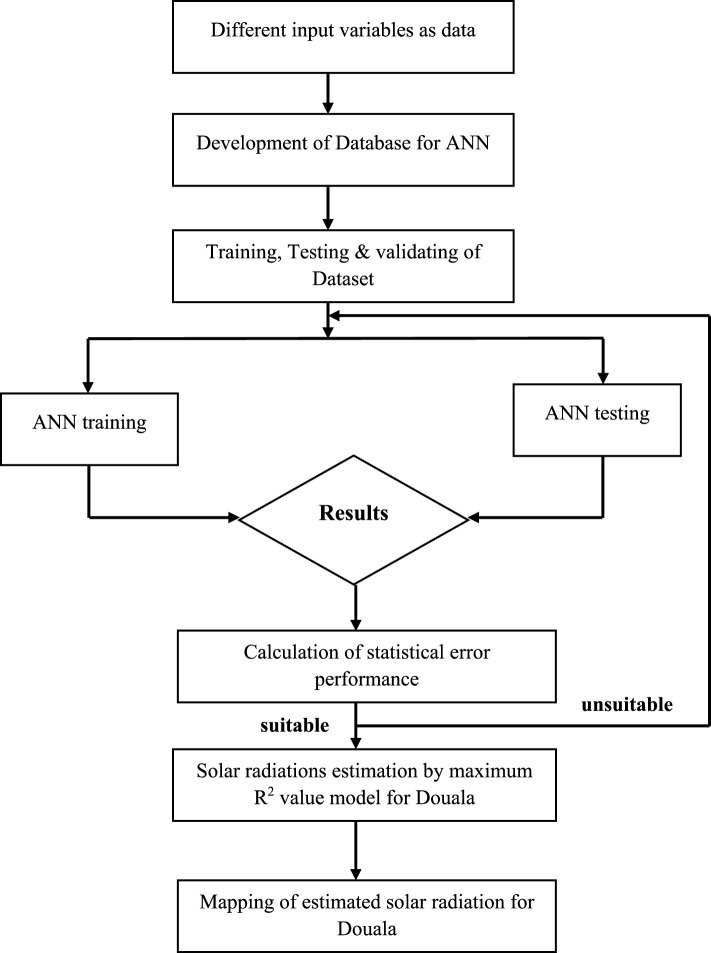
Flow diagram showing different steps to determine the ranking of parameter.

The system receives four input parameters, namely Temperature, Humidity, Atmospheric pressure, and Wind speed. The output of the system is the solar irradiance, which is the target data. The methodology employed in this investigation is depicted in Fig. 13. The initial step in developing the ANN model involved defining the input and output parameters. The location was determined using four characteristics as inputs, as described in the data collection process. In this study, an artificial neural network (ANN) model was developed by training and evaluating multiple multilayer perceptron (MLP) designs. The LM algorithm was utilized to train the feed-forward neural network. The network was trained using randomly allocated data. The Multilayer Perceptron (MLP) is trained using 80% of the available data, which corresponds to 11,024 samples. A portion of 15% of the data, equivalent to 2360 samples, is reserved for validation purposes. The remaining 5% of the data is used to test the models. The hidden layer's neuron count is adjusted and the network undergoes multiple training iterations to improve the obtained outcomes. The utilization of 6 inputs, 1 hidden layer, and 1 output layer has resulted in the occurrence of statistical error, as measured by the Mean Squared Error (MSE), across multiple experiments.

In our model validation and testing method, we have meticulously chosen precise proportions (80%, 15%, 5%) for training, validation, and testing, considering their importance in guaranteeing the precision and dependability of the model. These divisions are essential for the evaluation process since they enable a thorough review of the model's performance. To ensure successful learning and adaptation, we seek to give the model with a significant amount of information by assigning 80% of the data for training. By allocating 15% for validation, we may refine and improve the model, guaranteeing its ability to effectively apply to unfamiliar data. In addition, allocating 5% for testing allows us to comprehensively assess the model's performance on entirely novel data, so permitting well-informed judgments about its precision and capacity to generalize. The deliberate distribution of data for training, validation, and testing establishes a strong basis for assuring the model's resilience, improving its potential for practical use, and reinforcing the reliability of our results.

### Performance metrics of prediction accuracy

Two error assessment techniques, namely Mean Squared Error (MSE) and R-squared (R^2^), are utilized to evaluate the performance of the network and demonstrate the error rate of the proposed methods. The indicators are computed using Eqs. (11) and (12).

11 $${\text{MSE}}=\frac{1}{{\text{n}}}\sum_{{\text{i}}=1}^{{\text{n}}}({{\text{y}}}_{{\text{i}}}-\widehat{{\text{y}}}{)}^{2}$$

12 $${{\text{R}}}^{2}= 1-\frac{\sum_{{\text{i}}=1}^{{\text{n}}}({{\text{y}}}_{{\text{i}}}-\widehat{{\text{y}}}{)}^{2}}{\sum_{{\text{i}}=1}^{{\text{n}}}({\widehat{{\text{y}}})}^{2}}$$

The equation relates to the values of actual and predicted global solar radiation, denoted as ${{\text{y}}}_{{\text{i}}}$, and $\widehat{{\text{y}}}$, respectively.

The Mean Squared Error (MSE) is a statistical metric utilized to evaluate the performance of a model. Its value is always non-negative, where zero represents the optimal case. The coefficient of determination (R2) provides insight into the quality of the fit. Its range is from zero to one (0 ≤ R^2^ ≤ 1), with a higher value indicating a better-fit^80,81^.

## Results and discussion

**Figure 14:**
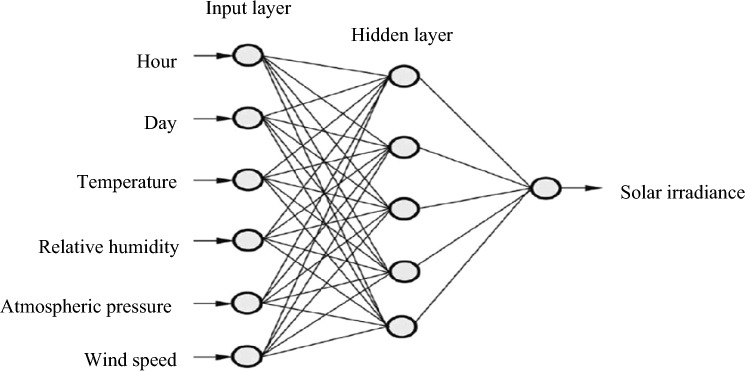
The suggested ANN model for solar irradiance estimation in the present work.

The present study involved the development of an artificial neural network (ANN) model to predict irradiance in the city of Douala, based on climate parameters. Multilayer back-propagation neural networks were designed and programmed using various architectures in accordance with standard practices. The meteorological station database of the IUT of Douala provided twenty-three months (January 2019–November 2020) of meteorological data for the purpose of training, testing, and validating the network. The network utilized four meteorological parameters, namely temperature, relative humidity, pressure, and wind speed, along with two temporal parameters, day and hour, as its inputs. The output of the network was the solar radiation intensity. The neural network model presented in Fig. 14 represents the optimal architecture as determined by the Artificial Neural Network (ANN) algorithm.

The computations are performed using MATLAB R2022a on a MacBook Pro equipped with a Core i5 processor clocked at 2.30Ghz and 8 GB of RAM. In order to assess the effectiveness of the developed artificial neural network (ANN) algorithms, two performance metrics were employed: the mean square error (MSE) and the coefficient of determination (R^2^). Following multiple iterations, an artificial neural network (ANN) consisting of three layers, including one input layer, one hidden layer, and one output layer, was selected. The tansig transfer function has been utilized for the hidden layer, while the linear transfer function (purelin) has been employed for the output layer. Multiple input combinations have undergone preliminary testing.

**Figure 15:**
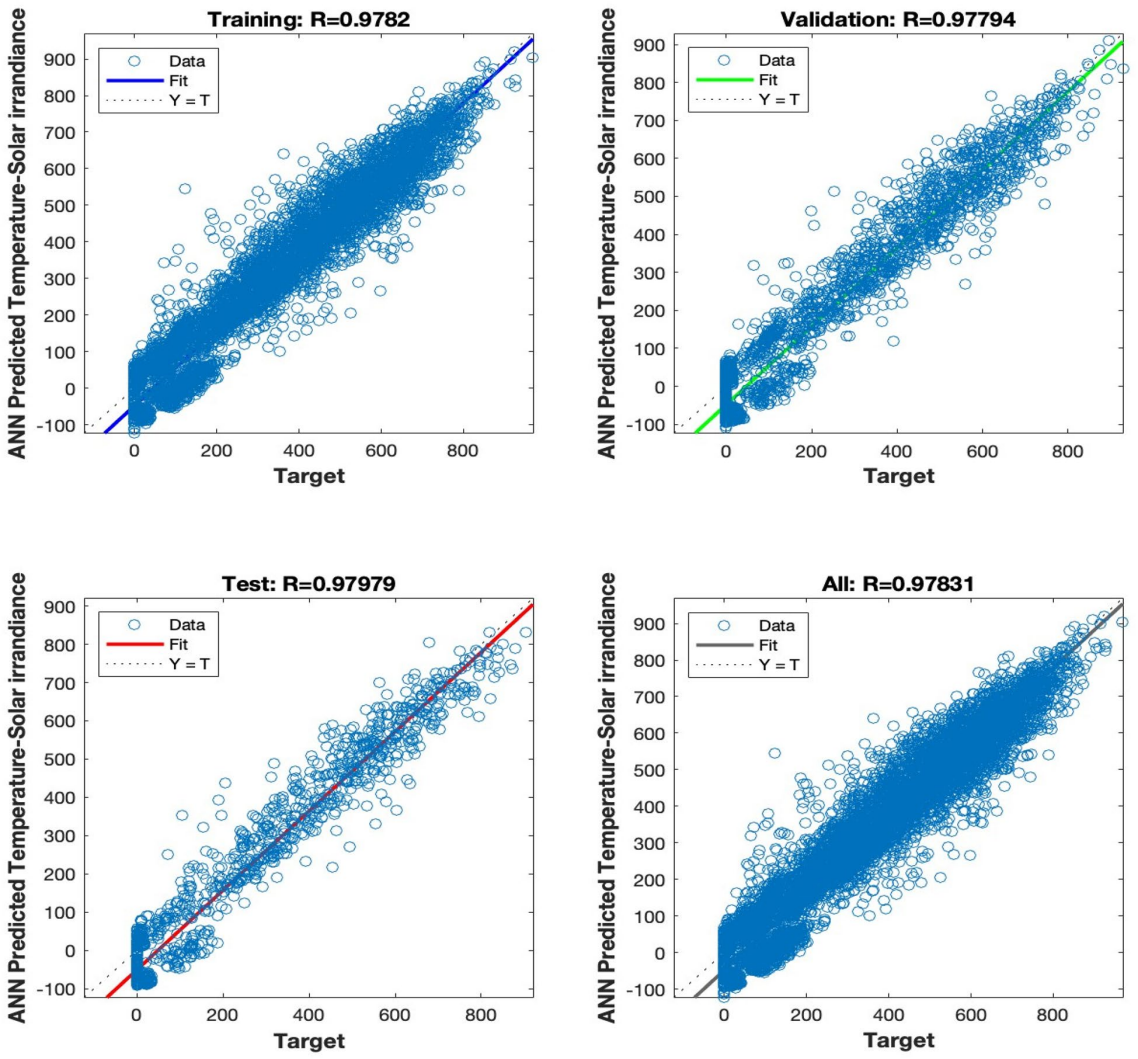
Regression plot for the ANN architecture of temperature data as input.

**Figure 16:**
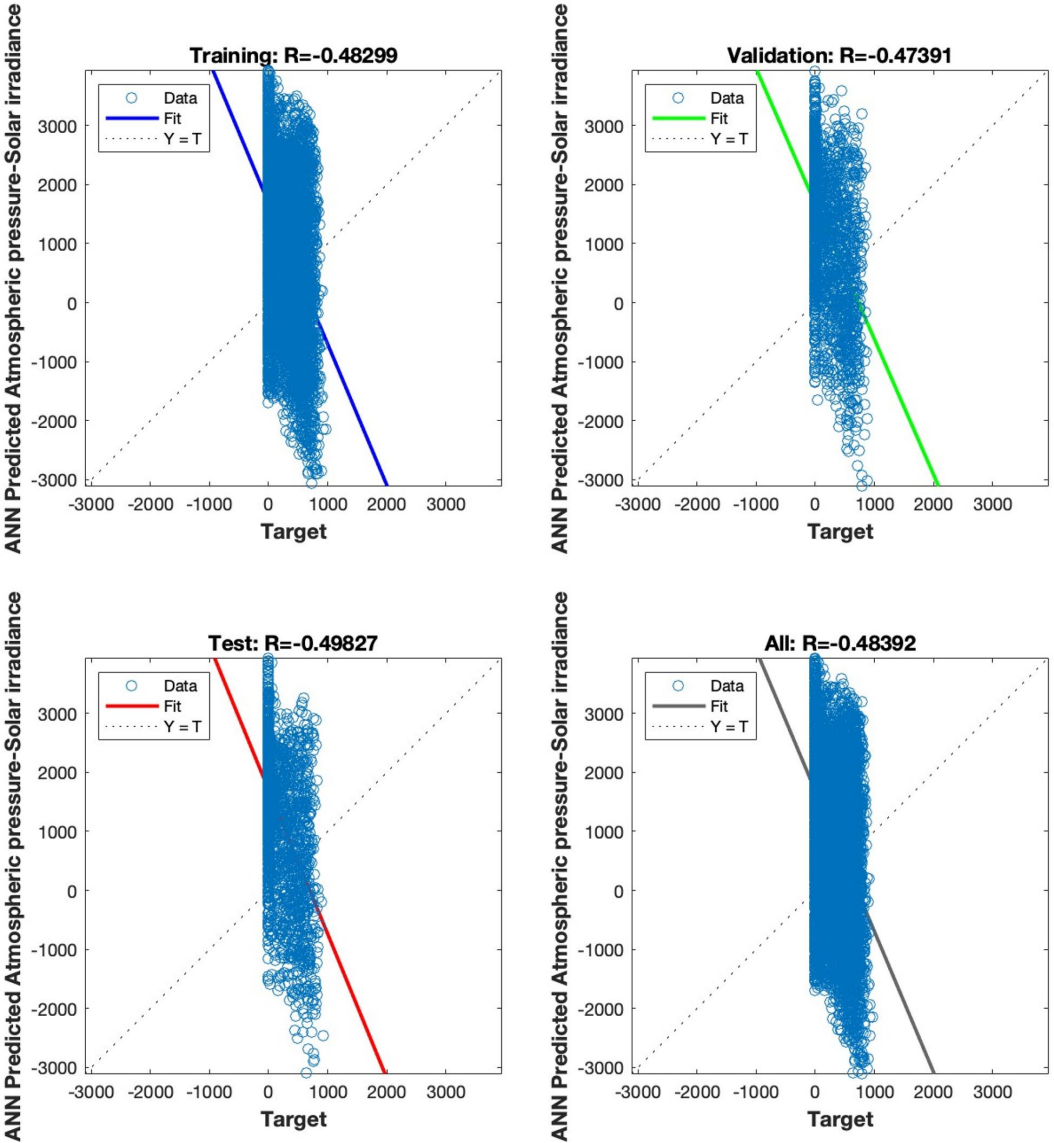
Regression plot for the ANN architecture of Atmospheric pressure data as input.

**Figure 17:**
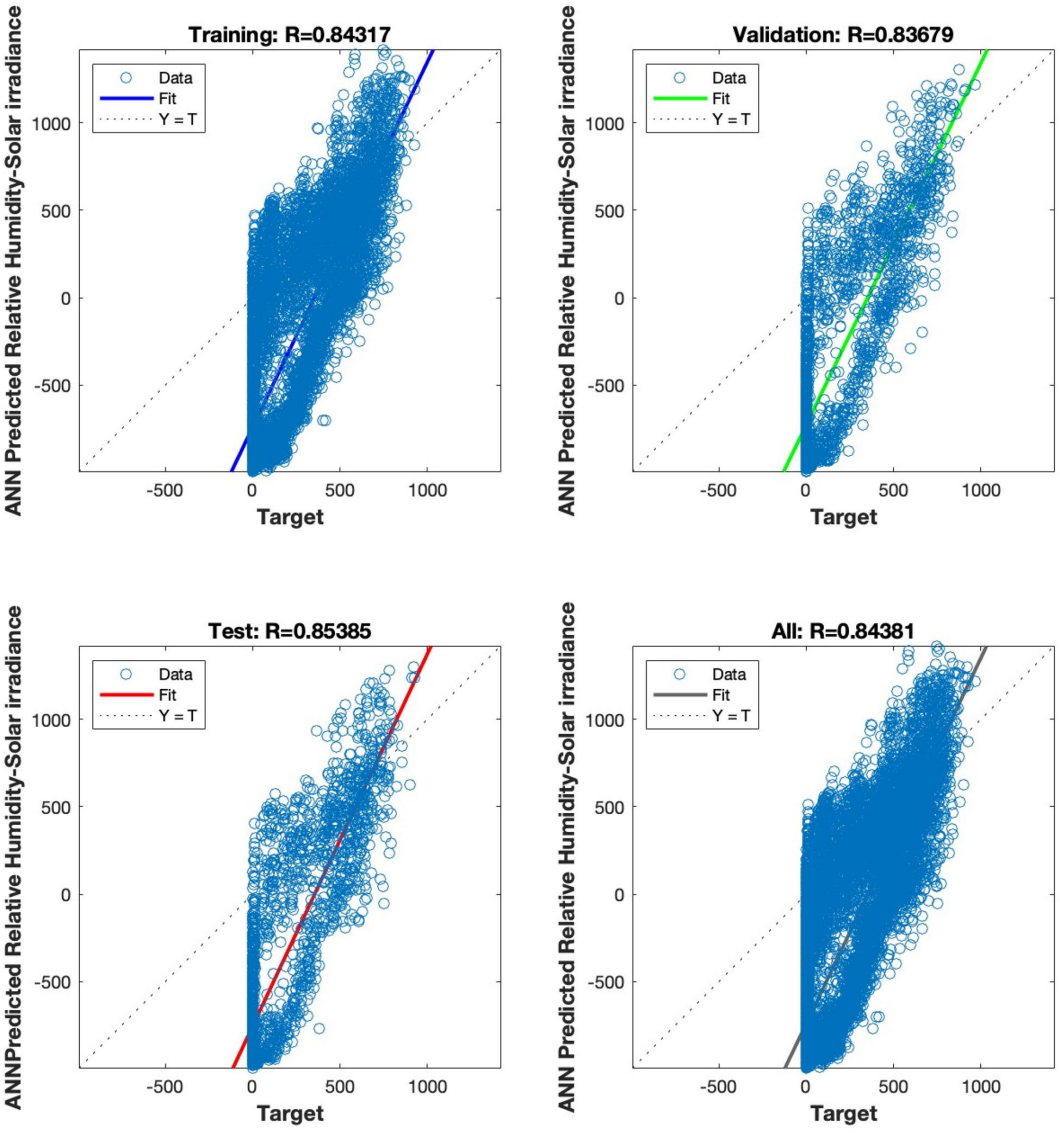
Regression plot for the ANN architecture of relative humidity data as input.

**Figure 18:**
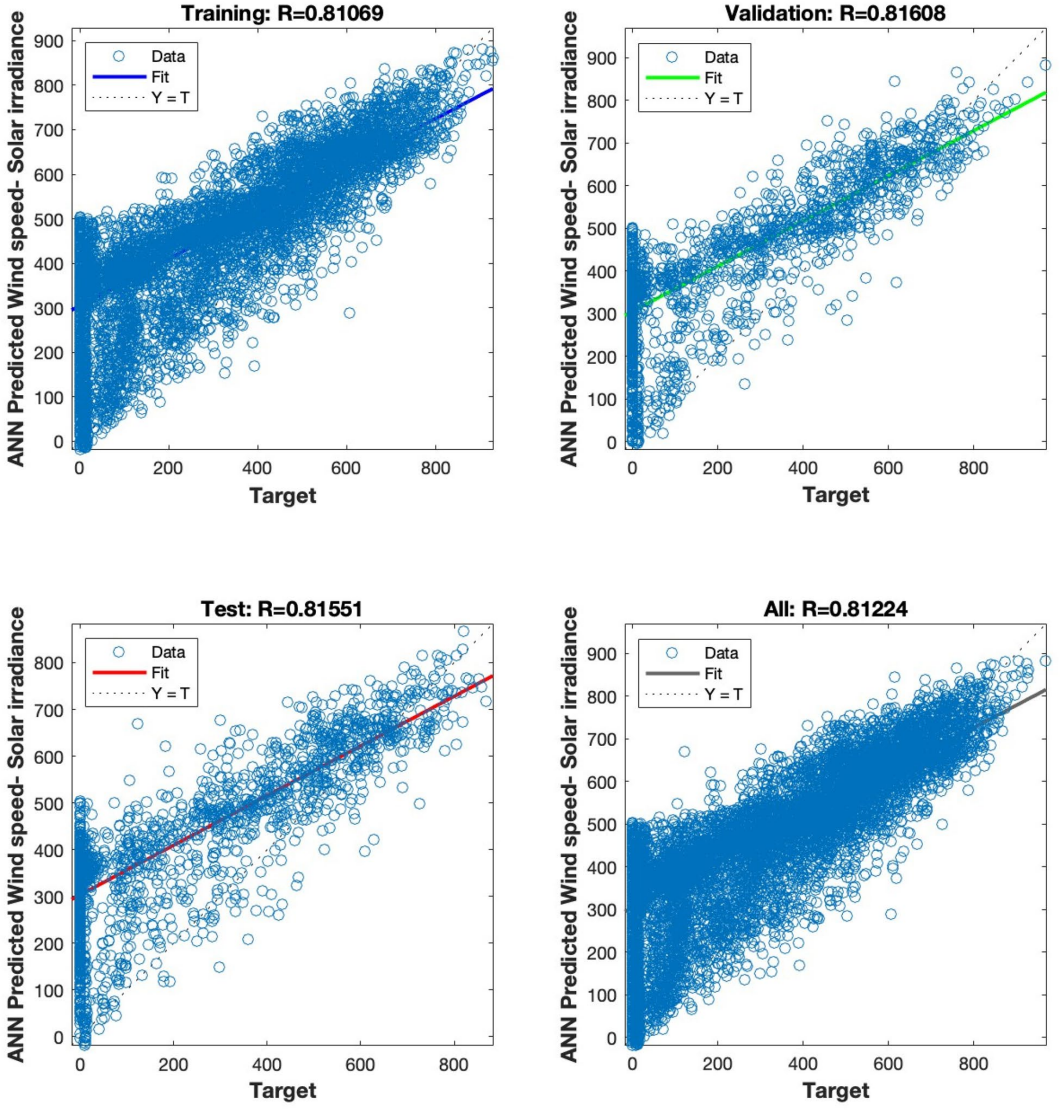
Regression plot for the ANN architecture of wind speed data as input.

**Figure 19:**
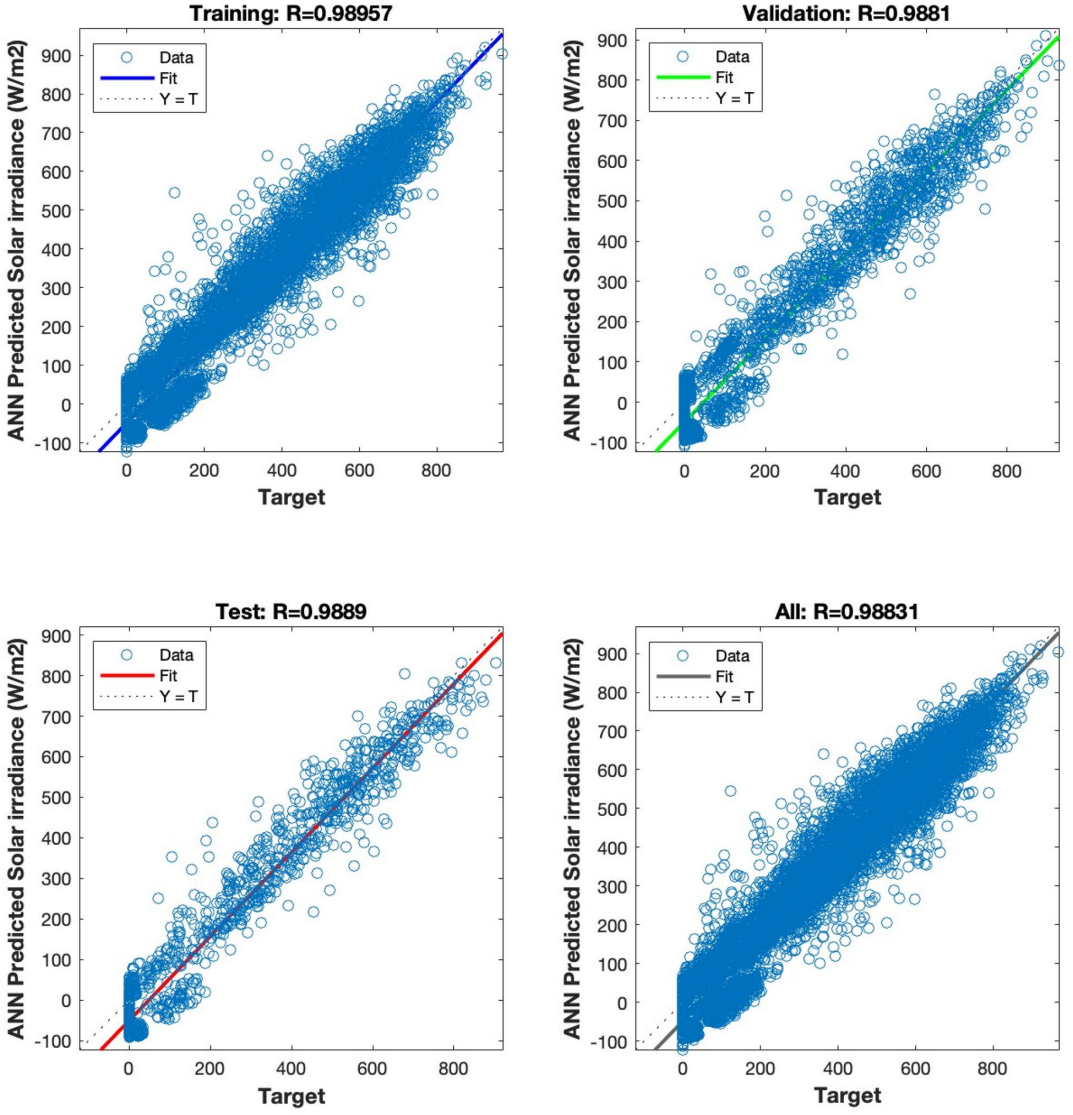
Regression plot for the ANN architecture for all combinations of data as input.

The regression analysis depicted in Figs. 15, 16, 17, 18 and 19 compares the predicted value to the target value. In this analysis, the output is considered the dependent variable, while the target is the independent variable. The purpose of this analysis is to evaluate the performance of the MLP. The correlation coefficient is a metric that indicates the degree of proportionality between the output and the targets. In order to achieve optimal performance of the MLP, it is desirable for this value to be close to unity.

Figures 15, 16, 17 and 18 illustrate the use of a solitary parameter as an input variable for forecasting solar irradiation. The temperature, atmospheric pressure, relative humidity, and wind speed variables exhibit correlation coefficients of 0.97831, 0.48392, 0.84381, and 0.81224, respectively, according to the data.

The correlation coefficient in Fig. 18 is 0.98831. The achieved value of 0.98831 provides empirical support for the efficacy of the suggested methodology. The results demonstrate that the artificial neural network (ANN) accurately predicts irradiance levels and nearly matches the actual values in all data sets.

The use of the four aforementioned criteria as inputs for Solar irradiance predictions has resulted in improved results. Table 1 presents a summary of the findings achieved for the most effective combinations of Artificial Neural Network (ANN) topologies throughout the training, testing, and validation stages, for varying quantities of neurons in the hidden layer.

**Table 1:** Results for optimal combinations of ANN.

Neurons of hidden layer	Accuracy	Levenberg–Marquardt		
		Train	Valid	Test
10	MSE	56,188	54,824	57,794
	R^2^	0.7306	0.7364	0.7206
20	MSE	6644	6687	6544
	R^2^	0.94109	0.9423	0.9027
50	MSE	**1194**	**1396**	**1427**
	R^2^	**0.98957**	**0.9881**	**0.9889**
100	MSE	1374	1402	1606
	R^2^	0.98722	0.9863	0.9853
200	MSE	11,605	12,728	12,047
	R^2^	0.9179	0.9081	0.916

Significant values are in bold.

**Figure 20:**
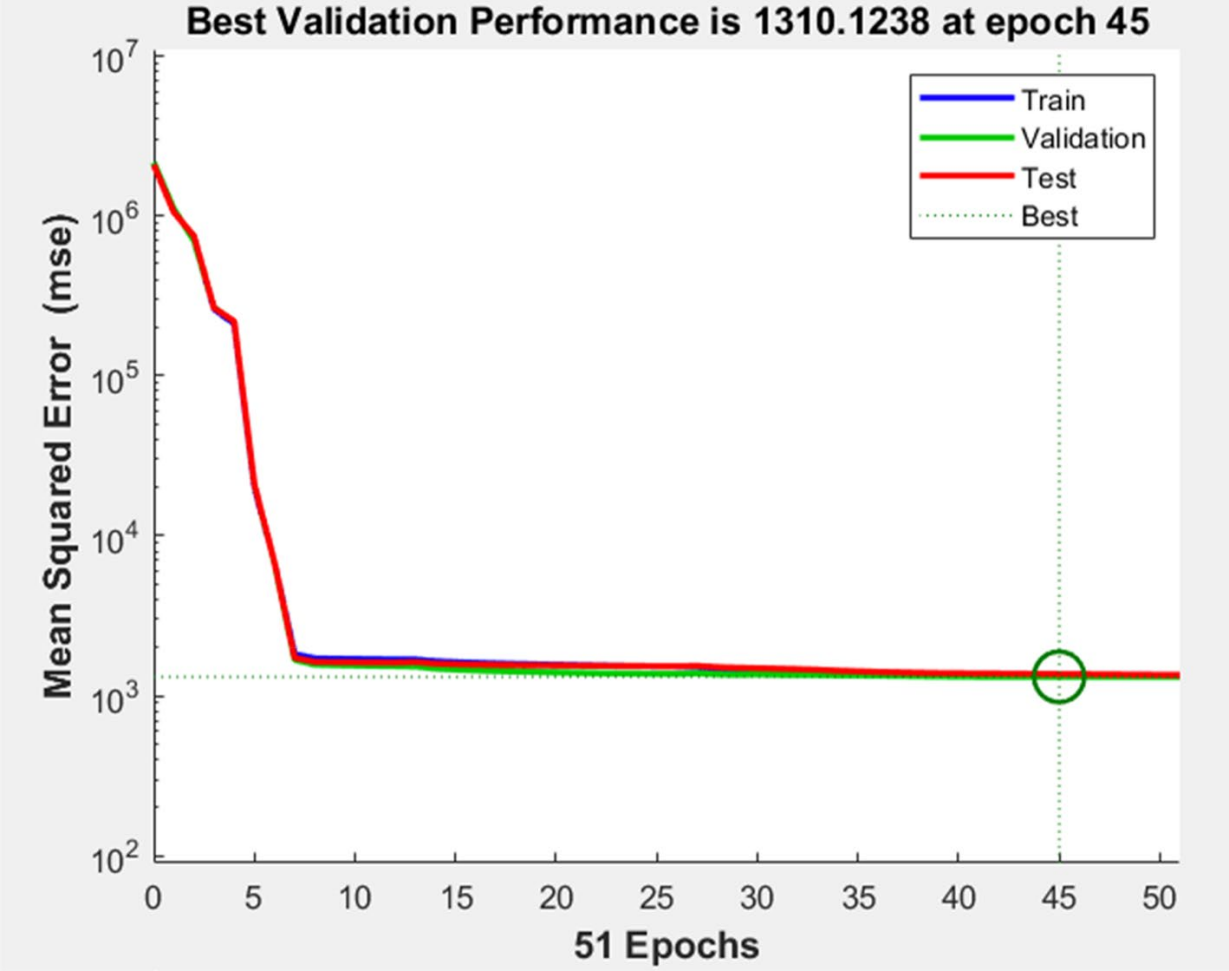
Neural network training performance.

Figure 19 is a representation of the convergence plot for the 6-50-1 equation. According to the convergence plot, there is a link that is inversely proportional between the mean square error and the number of epochs. It is important to note that the number of epochs reduces in proportion to the mean square error rises. Through the use of the convergence plot, it is shown that the testing and validation procedures have similar characteristics. At the 45th iteration (epoch), the Levenberg–Marquardt algorithm produced the best results in terms of computational efficiency as shown in Fig. 20. The period that had the least amount of validation mistake was the one that produced the best results. The performance of the neural network is enhanced by the combination of the values obtained during training, validation, and testing tests.

**Figure 21:**
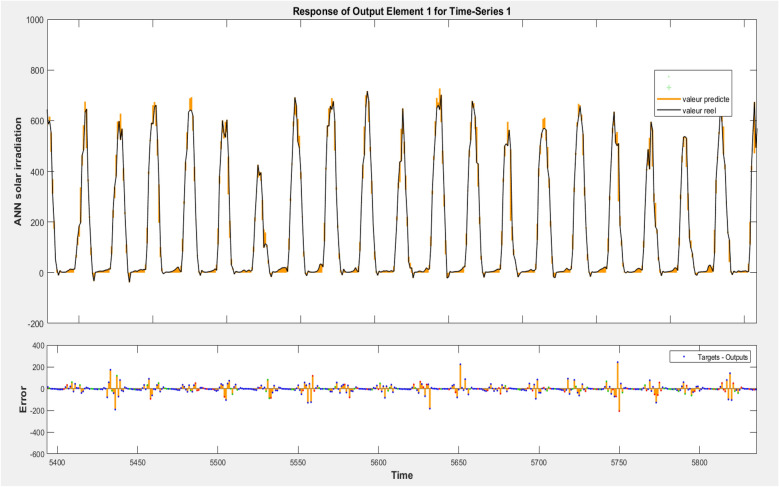
Measured and forecasted solar irradiance using temperature, wind speed, atmospheric pressure, and relative humidity.

The sun irradiance that was actually observed is contrasted with the solar irradiance that was projected by the artificial neural network by utilizing the air temperature, relative humidity, wind speed, and atmospheric pressure as inputs. This comparison is shown in Fig. 21. This image makes it very evident that it is possible to reach agreements between the two series that are suitable to both parties. Furthermore, it is clear that there is a significant connection between the data that were predicted and the data that were really obtained. This is something that we can see.

## Conclusion

Prediction using artificial intelligence, and especially using multilayer MLP neural networks, is of critical relevance in the study of Machine Learning, with several practical applications in areas as diverse as social networks, financial markets, navigation, and even meteorology. The many applications of prediction models are highlighted by their capacity to foretell consumer tastes, track social ties, and predict market movements, climate, and navigational paths.

This research focused on the use of neural network methods to predict hourly solar irradiance, which is crucial for the production of electricity from photovoltaic (PV) sources. We investigated the viability of employing a multilayer MLP neural network for solar irradiance forecasting by using meteorological information as inputs.

We conducted extensive experiments with various input configurations and found that a neural network trained with 50 hidden layer neurons, the logistic Sigmoid function, and four inputs (temperature, atmospheric pressure, relative humidity, and wind speed) produced the best results. An excellent 98.83% correlation was attained between observed and predicted sun irradiance using this methodology.

Our results provide strong evidence for the viability of the proposed model for calculating solar irradiance intensities at the Douala site and in other places with similar climatic circumstances.

This study illustrates the viability and efficacy of using artificial neural networks for precise solar irradiance forecasting and adds to the increasing body of knowledge in the area of solar energy forecasting.

Notwithstanding the exceptional performance, it is crucial to recognize potential constraints. An area that warrants additional investigation is the model's capacity to adapt to different environmental conditions and geographical locations. Moreover, the model's forecasting powers might be improved by using a wider range of meteorological data elements. Subsequent investigations may concentrate on using sophisticated feature engineering methods and investigating the incorporation of other environmental data, such as cloud cover and air pollution indices, to enhance the precision of solar irradiance forecasts.

Our study findings have significant practical implications. Our research demonstrates the practicality of using neural networks for accurate solar irradiance prediction, hence enhancing the development of solar energy forecasting methodologies. The consequences pertain to decision-making procedures for the execution of solar energy initiatives, not just in Central Africa but also globally. The use of precise solar irradiance predictions has the potential to improve the efficiency and dependability of solar energy generation. This not only benefits local energy policies but also supports worldwide renewable energy programs.

Our research may help guide decision-making and facilitate the effective implementation of solar energy projects in Central Africa and worldwide as renewable energy sources, especially photovoltaic systems, gain momentum in solving energy deficiencies.

## Data Availability

The datasets used and/or analysed during the current study available from the corresponding author on reasonable request. The data that support the findings of this study are available from the corresponding author upon reasonable request.
