# Author Correction: A multilayer perceptron neural network approach for optimizing solar irradiance forecasting in Central Africa with meteorological insights

**DOI:** 10.1038/s41598-024-55883-z

**Published:** 2024-03-04

**Authors:** Inoussah Moungnutou Mfetoum, Simon Koumi Ngoh, Reagan Jean Jacques Molu, Brice Félix Nde Kenfack, Raphaël Onguene, Serge Raoul Dzonde Naoussi, Jean Gaston Tamba, Mohit Bajaj, Milkias Berhanu

**Affiliations:** 1 Technologies and Applied Sciences Laboratory, University Institute of Technology of Douala, University of Douala, P.O. Box: 8689, Douala, Cameroon; 2 Department of Industrial Engineering and Maintenance, University Institute of Technology of Douala, University of Douala, P.O. Box: 8689, Douala, Cameroon; 3 Department of Thermal Engineering and Energy, University Institute of Technology of Douala, University of Douala, P.O. Box: 8689, Douala, Cameroon; 4 Transport and Applied Logistic Laboratory, University Institute of Technology of Douala, University of Douala, P.O. Box: 8689, Douala, Cameroon; 5 Department of Electrical Engineering, Graphic Era (Deemed to be University), Dehradun, 248002 India; 6 Hourani Center for Applied Scientific Research, Al-Ahliyya Amman University, Amman, Jordan; 7 Graphic Era Hill University, Dehradun, 248002 India; 8 Applied Science Research Center, Applied Science Private University, Amman, 11937 Jordan; 9 Department of Electrical and Computer Engineering, College of Engineering, Addis Ababa Science and Technology University, Addis Ababa, Ethiopia

Correction to: *Scientific Reports* 10.1038/s41598-024-54181-y, published online 12 February 2024

In the original version of this Article Milkias Berhanu was incorrectly affiliated with ‘Technologies and Applied Sciences Laboratory, University Institute of Technology of Douala, University of Douala, P.O. Box: 8689, Douala, Cameroon’. The correct affiliation is listed below:

Department of Electrical and Computer Engineering, College of Engineering, Addis Ababa Science and Technology University, Addis Ababa, Ethiopia

The original Article has been corrected.

